# Identifying Research Priorities to Promote the Well-Being of Family Caregivers of Canadians with Intellectual and/or Developmental Disabilities: A Pilot Delphi Study

**DOI:** 10.3390/ijerph20227072

**Published:** 2023-11-16

**Authors:** Afolasade Fakolade, Caitlin Stone, Nicole Bobbette

**Affiliations:** Louise D. Acton Building, School of Rehabilitation Therapy, Queen’s University, 31 George Street, Kingston, ON K7L 3N6, Canadanicole.bobbette@queensu.ca (N.B.)

**Keywords:** caregivers, developmental disabilities, Delphi technique, research agenda, wellness

## Abstract

Current programming and resources aimed at supporting the well-being of family caregivers often fail to address considerations unique to those caring for people with intellectual and/or developmental disabilities (IDDs). As a result, many caregivers of people with IDD feel isolated, stressed, and burnt out. A targeted research agenda informed by key stakeholders is needed and would allow research teams to coordinate resources, talents, and efforts to progress family caregiver well-being research in this area quickly and effectively. To address this aim, this pilot study used a Delphi design based on 2 rounds of questionnaires. In round 1, 19 stakeholders (18 females, 1 male), including 12 family caregivers, 3 rehabilitation providers, 2 researchers, and 2 organizational representatives, identified broad areas for caregiver well-being research. After collating the responses from round 1, stakeholders were asked to rank whether each area was considered a research priority in round 2. Data were analyzed using descriptive statistics and conventional content analysis. Eighteen stakeholders completed the round 2 survey (1 caregiver did not complete the round 2 survey), after which a consensus was reached. Stakeholders identified nine broad priorities, including system-level programs and services, models of care, health promotion, social inclusion, equity and diversity, capacity building, care planning along the lifespan, and balancing formal and natural community-based supports. Although preliminary in nature, the research priorities generated using an inclusive and systematic process may inform future efforts to promote the well-being of caregivers of Canadians with IDD.

## 1. Introduction

Family caregivers (i.e., relatives or friends) play a critical role in supporting persons living with complex chronic conditions and disabilities to ensure their basic needs are met while their rights are respected and protected [1,2]. Over 8 million Canadian adults (25% of Canadians) provide unpaid family caregiving for a person living with complex chronic conditions and disabilities [3], such as intellectual and developmental disabilities (IDDs). IDD is a broad term that includes conditions that are identified within the developmental period (<22 years), are lifelong in nature, and include impairments in cognitive and/or adaptive functioning (i.e., conceptual, social, and practical skills). Health conditions that are often included under the umbrella term are intellectual disability (ID), Down’s syndrome, autism spectrum disorder (ASD), attention deficit hyperactivity disorder (ADHD), and other specific communication/learning and motor disorders [4]. It is important to acknowledge that notable differences exist between these conditions, and having a developmental disability does not necessarily mean you also have a cognitive impairment. Multiple terms have been used to describe the range of conditions associated with IDD; however, IDD is a term recognized by key international groups (e.g., the American Association on Intellectual and Developmental Disabilities and the International Association for the Scientific Study of Intellectual and Developmental Disabilities) and is used in this study.

The type of support provided by family caregivers of persons with IDD varies based on the age, health condition, and needs of the care recipient [5]. Family caregiving support can range from instrumental (e.g., transportation, care coordination, and healthcare-related tasks), personal care (e.g., feeding and bathing), and general social and emotional support [6]. Without family caregiving support, many people with IDD will be unable to live, play, work, or participate in activities at home and in the community [3,7]. The presence of one or more family caregivers also makes it possible for some people with IDD to delay or avoid crisis situations that may result in hospitalizations or even entry into long-term care [7]. Family caregiving is a multidimensional experience wherein caregivers may derive value and positive benefits from providing care and simultaneously be vulnerable to the role’s negative physical, psychological, financial, and social impacts [8]. Providing ongoing support for people with IDD is associated with high stress levels, poor physical and psychological outcomes, and increased risk of morbidity and mortality [9,10,11,12]. Family caregivers of people with IDD report negative impacts on employment and income, exacerbating financial difficulties in providing care, and leading to substantial economic costs for families [13,14]. The social dimensions of the challenges experienced by caregivers of people with IDD also need to be acknowledged. Caregivers often report feelings of social isolation, loneliness, reduced participation in social activities, and declines in social support over time [8,15]. Many family caregivers continue to report unmet needs and substantial burdens, and often struggle to cope with their caregiving responsibilities [16].

In recent years, there have been calls for urgent systemic attention to the rights, needs, and demands of persons with disabilities, including those living with IDD [17]. Resultingly, a person with IDD is understandably often the focus of most health and social care research in this field. However, in the context of developing appropriate structures to support the families of people living with IDD (inclusive of the persons with IDD themselves), the well-being of family caregivers also deserves considerable attention. Family caregivers are considered the backbone of social care provision [18], and their well-being is inter-dependent on the well-being of their care recipients, underscoring the need to advance knowledge about how best to optimize disability-related supports for families of people with IDD [19,20,21]. In Canada, support mechanisms, including formal support delivered through paid providers (psychiatrists, social workers, rehabilitation providers, etc., in integrated health teams) [22] or a combination of provincial, territorial, and federal public benefit schemes (Federal Child Disability Benefit, Canada Caregiver Credit, Ontario Assistance for Children with Severe Disabilities, etc.), have been developed [23]. These mechanisms vary in format, model, and scale to alleviate caregiver burden and improve well-being. However, family caregivers of people with IDD continue expressing concerns and unmet needs [16,24]. For instance, existing support mechanisms primarily focus on the needs of the person with IDD, are means-tested and fragmented, and often fail to consider the diversity of caregiving or the complexity of caregiver needs [25]. This is unfortunate, as poor access to adequate support negatively impacts caregivers’ well-being, increasing the burden of disability for individuals, families, and society [26]. Certain social groups of caregivers are particularly vulnerable, for example, Indigenous Peoples and ethnic minorities, sexual and gender minorities, and other socially marginalized groups [27]. The root causes of this vulnerability lie in a combination of factors, including geographical locations, financial, socio-economic, cultural, and gender status, and access to resources, services, decision-making power, and justice. In this sense, the wellness experiences and challenges reported by caregivers are not isolated to the individual or personal sphere but situated against a backdrop of social dimensions of health at multiple levels that require concerted efforts to address.

The scientific community, through research, has a significant potential to make important contributions to caregiver well-being by generating evidence to foster action and systems change [25]. However, many research teams from different disciplinary backgrounds are engaging in overlapping or complementary studies rather than working in a coordinated way that could accelerate progress [28]. To realize our potential for impact, research teams must identify pathways to combine resources, talents, and efforts to progress research and advocacy efforts on family caregiver well-being quickly and effectively. Researchers must build high-quality evidence and use the evidence to inform strategies and advocacy efforts so that caregivers of Canadians with IDD are getting the best support possible. These activities require the development of shared goals and research priorities. Yet, there remains very little data regarding what caregivers of Canadians with IDD and other stakeholders (healthcare providers, support organizations, etc.) believe are the most critical priorities for caregiver well-being research [29,30].

A research agenda will take the first steps in developing shared goals and priorities to ensure that the research on family caregiver well-being is coordinated, sustainable, and focused on concrete needs. A shared research agenda focusing on enhancing the well-being of caregivers of Canadians with IDD will allow individual researchers and research teams to focus their efforts on priority areas, ultimately increasing the likelihood of making the most significant impact possible [31]. Furthermore, funding agencies and decision-makers can use the research agenda to improve resource allocation and discovery, particularly in the current environment of highly competitive and minimally available research funding [32]. Once created, a shared research agenda is an invitation to researchers and funders to support them in conducting research that addresses areas of importance to caregivers. It will also help to reduce unnecessary research initiatives that do not improve existing evidence in this field [33]. Therefore, the purpose of this Delphi design pilot study was to develop a list of the top priorities to guide research targeting the well-being of caregivers of Canadians with IDD. The specific research question was what do family caregivers, healthcare providers, researchers, and representatives of organizations that provide support for families of persons with disabilities deem are the priority areas for caregiver well-being research in IDD in Canada?

## 2. Materials and Methods

### 2.1. Recruitment and Enrolment

There is no power analysis in the Delphi method; instead, the representativeness of the results is based on the quality of the panel rather than on its size [34,35]. Nevertheless, a minimum of 12 participants from heterogeneous groups (e.g., experts from different disciples) is recommended in the literature to provide a range of perspectives and to improve the reliability of the findings [36,37]. With these considerations in mind, our aim was to recruit a non-probability purposive sample of at least 12 participants from four stakeholder groups. The stakeholder groups were chosen to maximize caregiver, professional, and scientific community engagement [38]. They included family caregivers, rehabilitation providers, researchers, and representatives of organizations that provide support for Canadians with IDD and/or their caregivers.

Caregivers and organizational representatives were recruited using study flyers distributed via the online newsletters of relevant support organizations across Canada (e.g., Siblings Collaborative/Siblings Canada, Carers Canada). Rehabilitation providers and researchers were recruited through direct emails to the research team’s clinical contacts and the corresponding author(s) on recent journal articles related to caregiving for people with IDD.

Regardless of the recruitment method, interested individuals were asked to contact the research team to indicate their interest in participating in the study. When individuals contacted the research office, a research assistant emailed them a letter of information about the study and a personalized electronic invitation survey to complete the first round of questioning. When individuals activated the electronic invitation, they were guided to the survey website, where they were again presented with detailed information about the purpose of the study and a consent form. They were then invited to undergo online screening through the survey site to confirm their eligibility. Consenting individuals who met the eligibility criteria were enrolled and could complete the round 1 survey. The survey was automatically terminated for those who did not indicate consent or meet the eligibility criteria.

### 2.2. Inclusion Criteria

To be included in the study, family caregivers had to be (1) ≥18 years old and (2) provide ≥1.0 h/day of unpaid assistance/help to an adult with IDD. IDD was described as conditions that are identified within the developmental period (<22 years), are lifelong in nature, and include impairments in cognitive and/or adaptive functioning (i.e., conceptual, social, and practical skills). Examples of health conditions that were included under the umbrella term were ID, Down’s syndrome, ASD, ADHD, and other specific communication/learning and motor disorders. Rehabilitation providers, researchers, and organizational representatives had to have ≥2 years of experience supporting people with IDD, publishing caregiver well-being research, or working/volunteering in an organization that provides support services for people with IDD and/or their caregivers. All stakeholders were required to have access to an internet-enabled device (i.e., computer, tablet, or phone) to participate in the study.

### 2.3. Data Collection

A Delphi survey study was conducted. The Delphi technique is a systematic approach for achieving consensus on priorities through multiple rounds of survey questioning with relevant stakeholders [39]. Key features of the Delphi technique include stakeholder anonymity throughout a series of iterative questionnaire rounds and controlled feedback between rounds. Details of collective group opinion allow stakeholders to retain or change their earlier opinions in light of other views. This study used a Delphi survey because the technique can facilitate decisions for recommendations in areas with a paucity of knowledge [40], such as caregiver well-being research priorities, and has been used successfully to develop research priorities in various health-related contexts [29].

In a classic Delphi design, the first survey round consists of qualitative open-ended questions to establish the breadth of stakeholders’ opinions on the topic. This round allows each stakeholder to contribute information they believe is important in answering the questions posed [41]. The second survey round is developed from the items identified in round 1, and used to determine areas of agreement and disagreement among stakeholders. This round focuses on achieving consensus on the items derived from round 1 [42]. Thus, in the first round of the present Delphi survey study, all stakeholders were asked to identify broad topics and areas for caregiver well-being research, including brief descriptions and rationales for each area. In addition, demographic information, including evidence of relevant expertise, was captured. Personalized reminder emails were sent if stakeholders had not completed the survey within the stipulated timeframe. All stakeholders who completed the round 1 survey received a personalized email link to the round 2 online survey, their previous responses, and a summary of the group responses from the round 1 survey, including a description of each research area. They were then asked to prioritize each research area, using a 5-point scale (1 = “not important to conduct research in this area” to 5 = “very important to conduct research in this area”). Each survey round took ~30 min and was open for two weeks. During each round, participants were invited to provide further comments regarding each identified area. The round 1 survey was completed between July and September 2022, while the round 2 survey was conducted between October and November 2022. All surveys were completed via an online website, Qualtrics (Qualtrics, Provo, UT, USA), allowing for individual and anonymous responses [43]. Participants received CAD75 in online gift cards at the end of round 2 data collection.

### 2.4. Data Analysis

Data analysis occurred after each survey round. Data were downloaded from Qualtrics and imported into IBM SPSS Statistics for Windows version 28.0 (IBM Corp., Armonk, NY, USA) for analysis. Stakeholders’ characteristics and group responses were summarized using descriptive statistics (n, %, and mean/SD, as appropriate). A conventional content analysis approach [31] was applied to the free-text research descriptions and rationales provided in round 1. Content analysis is the most commonly used process to analyze Delphi round 1 survey data [34,44]. Furthermore, content analysis was considered an appropriate analytic strategy given the exploratory nature of the research and the aim to generate and describe broad priority areas for caregiver well-being research in IDD in Canada. Specifically, the three authors independently read the free-text responses from three randomly selected participants to familiarize themselves with the data. Then, the second author (CS) began highlighting text that appeared to describe potential topics for research and writing down a keyword/phrase that summarized these topics, using participants’ words. After this initial inductive open coding, the research team met to develop labels for preliminary codes that capture potential research areas. The second author then coded the remaining surveys (and re-coded the original surveys) using these codes and adding new codes when she encountered data that did not fit into an existing code. Another research team meeting occurred after the second author completed the coding of the remaining surveys. During this meeting, the three authors merged the codes that overlapped highly into categories, describing broad research areas, in preparation for the round 2 survey.

Although consensus should be defined a priori, there is no single agreed-upon method for determining consensus in Delphi studies [34]. However, a threshold of 70% is commonly used to promote the validity of the findings [45,46]. Therefore, in this study, consensus was defined, a priori, as ≥70% of participants selecting a rating of “important” or “very important” to conduct research in this area. Items achieving ≥70% consensus in round 2 were included in the final list of research priority areas.

## 3. Results

### 3.1. Participants

Of the 43 stakeholders invited to participate in the study, 5 declined and 13 did not respond to the invitation. Twenty-five individuals agreed to participate and received the electronic invitation to complete the round 1 survey. After receiving this invitation, six individuals declined to participate in the study and did not activate the survey link. Nineteen (18 female, 1 male) of the 43 invited individuals were enrolled in the study and responded to the round 1 survey (44% response rate). The stakeholders included 12 family caregivers who spent 7.7 ± 5.1 h/day providing care. Caregivers had been providing care for a mean of 24. 6 ± 9.9 years. Of the 12 caregivers, 7 were co-habituating with their care recipients. Caregivers were providing care for care recipients with Down’s syndrome (n = 1), cerebral palsy (n = 1), ASD (n = 2), ID co-occurring with other conditions such as Epilepsy, Alzheimer’s disease, and Huntington’s disease (n = 8). Three rehabilitation providers, two researchers, and two organizational representatives with 10.3 ± 4.5, 17.5 ± 10.6, and 12.0 ± 11.3 years of experience, respectively, were enrolled. Eighteen stakeholders responded to the round 2 survey (42% response rate). One family caregiver was lost at follow-up.

### 3.2. Survey Findings

**Figure 1:**
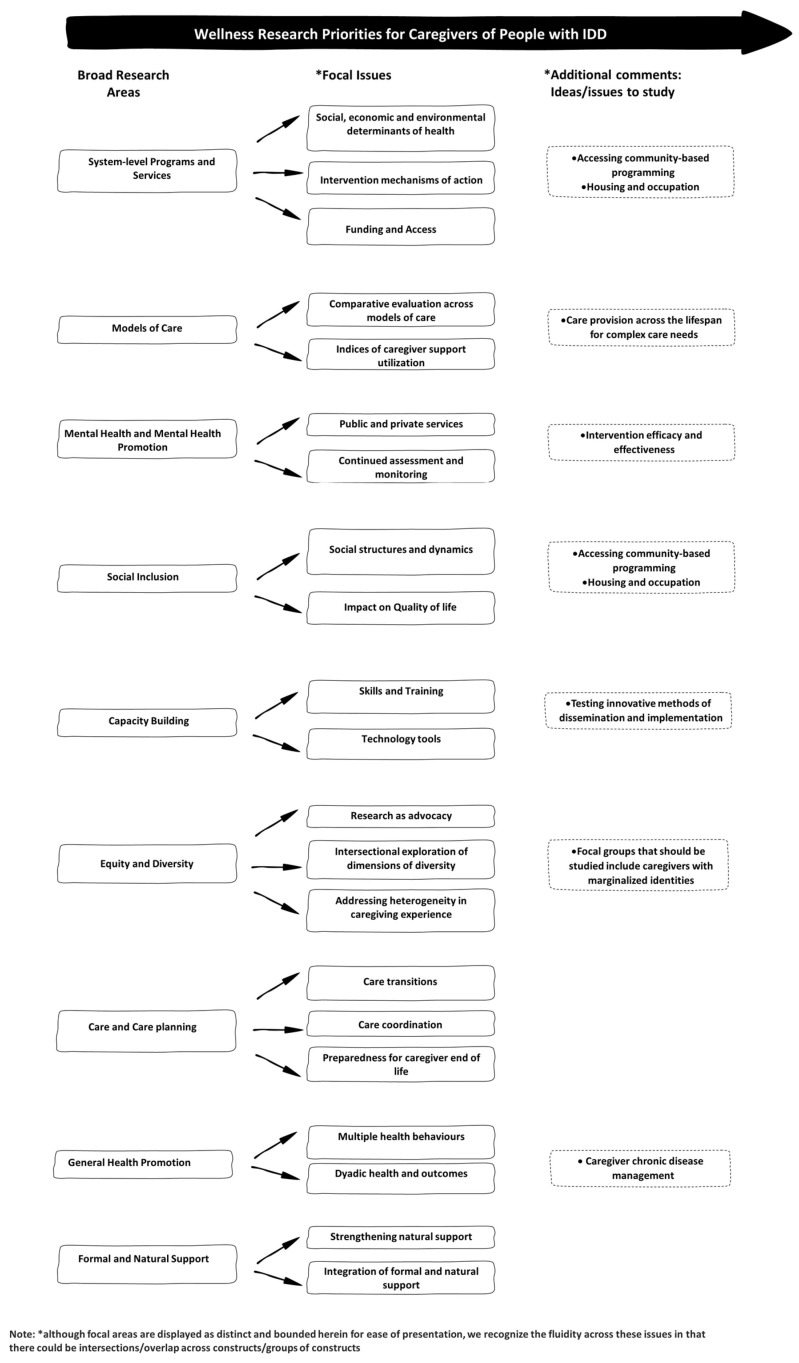
A summary of the nine research areas.

The distribution of responses according to the stakeholder group is shown in Figure S1. Participants identified 112 potential research areas/topics in round 1 (see Table S1), which were grouped into nine broad areas for ranking in round 2. Using the a priori criterion for defining consensus, eight research topics reached a consensus as priority areas in round 2 (i.e., 70% or more participants rated the topic as “important” or “very important”). Of note, there was near consensus (67%) on the ninth research area, which was subsequently retained. Based on the high level of consensus in round 2 and the risk of a potentially low response rate in the third round, a third round was not conducted. The nine research areas are presented in Figure 1 and discussed below.

#### 3.2.1. System-Level Programs and Services (94%)

Participants identified a need to explore how social, economic, and environmental determinants of health impact the well-being of caregivers supporting Canadians with IDD. Participants further noted that ongoing challenges with current system-level programming and services directly impact care recipients’ and caregivers’ individual and collective well-being. For example, participants expressed the challenge of having sufficient financial support to provide caregiving, cover necessary medical and life needs, access community-based programming, and limited housing, leisure, and/or employment opportunities for their care recipients. Research that examines the mechanisms by which interventions in these areas promote the health and well-being of caregivers is warranted.

#### 3.2.2. Models of Care (89%)

Participants acknowledged that to improve caregiving support in Canada, available models of care for caregivers nationally and internationally must be evaluated and compared. In particular, they noted the importance of exploring how caregivers utilize support, who provides the support and under what models of care, and what gaps in care provision continue to exist for caregivers of Canadians with IDD, particularly those with complex care needs (e.g., developmental disabilities co-occurring with other chronic health conditions, such as Epilepsy).

#### 3.2.3. Mental Health Promotion and Mental Healthcare (83%)

Participants stated that while mental health is receiving current research attention, access to and appropriateness of public and private-funded mental health care services for Canadians with IDD is limited. Systems to assess and monitor caregiver mental health must be developed to intervene before crisis situations emerge. Research must also evaluate the benefits of targeted mental health promotion interventions for caregivers and their care recipients.

#### 3.2.4. Social Inclusion (83%)

Participants noted the importance of research evaluating the impact of friendships, belongingness, community connectedness, and social inclusion on caregivers’ and care recipients’ health, well-being, and quality of life. Participants further identified the need for research to explore how supporting the autonomy and full participation of Canadians with IDD in their social networks impacts the collective well-being of caregivers and care recipients.

#### 3.2.5. Caregiver Capacity Building (78%)

Participants acknowledged that most caregivers assume the caregiving role without any preparation or the necessary tools to succeed. Therefore, there is a need to develop and evaluate interventions and innovative technology-supported tools and resources that enhance caregiver knowledge and skills to manage their caregiving responsibilities. Importantly, given the heterogeneity of needs in this population and the fact that caring will continue throughout the lifespan, there needs to be a suite of tools and resources that can be individualized and tailored for support. In addition, participants emphasized that innovative methods to promote implementation in practice are needed where existing efficacious interventions exist.

#### 3.2.6. Equity and Diversity in Care Provision (78%)

Participants noted the importance of research that contributes to dismantling the historical systems of inequality and oppression that continue to influence the health and well-being of caregivers of Canadians with IDD, particularly those with intersecting marginalized identities. Participants advocated for research that uses an equity, diversity, and intersectionality lens to explore how multiple dimensions of diversity, such as age, gender, race, (dis)ability, education, immigration status, and geography, interact with one another in the context of power and social inequities to inform the caregiving experience. Participants also emphasized that caregivers are not a homogeneous group, but each person possesses unique and complex features. Therefore, researchers need to recognize and incorporate an intersectionality perspective in a structured manner in the design and implementation of research studies.

#### 3.2.7. Care and Care Planning along the Lifespan (72%)

Participants acknowledged the profound challenges inherent in transitioning from adolescence to young adulthood, which are related to physiological, psychological, and social changes as young people orient toward greater independence in many concurrent areas of life. Many caregivers also raised the concern of who will care for their loved one when they are gone, acknowledging the transition into older adulthood for both the care recipient and caregiver. The call for research here is to explore how to improve lifelong care coordination while adequately preparing caregivers and care recipients for transitions in care and to identify services needed at different life stages to enhance the collective well-being of caregivers and care recipients.

#### 3.2.8. General Health Promotion (72%)

Participants emphasized the importance of acknowledging the unique inter-relatedness of health behaviors and outcomes among caregivers and their care recipients. Participants called for research that examines how caregivers’ and care-recipients’ health behaviors (e.g., sleep, nutrition, exercise), emotional health, pain management, and chronic disease management independently and collectively impact the ability to provide and receive care.

#### 3.2.9. Balance of Formal and Natural Community-Based Supports (67%)

While participants recognized that formal supports are typically prioritized in Canadian society, they also identified the need for research to inform the creation of community-based “wrap-around” support systems consisting of balanced formal (paid) and natural (unpaid/informal) support. It is necessary to explore how best to integrate available supports, as opposed to a dichotomy of only two options—formal vs. natural (organized, recurring, or infrequent unpaid support provided by a peer, friend, family member, or other support persons in the community). Further, participants identified a need to explore how integrating formal and natural supports impacts the well-being of caregivers.

#### 3.2.10. Caregiver Engagement in Research

Participants’ optional free-text responses primarily focused on the importance of conducting research in partnership and co-led with caregivers. Further, the participants highlighted the need for research that captures the stories of caregivers through qualitative methodologies, including but not limited to interviews, focus groups, and arts-based methods. The use of various online, in-person, or asynchronous intervention approaches is also recommended to optimize caregivers’ participation in this work. Finally, participants identified the need for implementation research to apply existing research findings to improve the health, functional status, and quality of life of families of Canadians affected by IDD (inclusive of persons with disabilities).

## 4. Discussion

This Delphi study aimed to engage a diverse group of experts in developing a comprehensive research agenda focused on promoting the well-being of caregivers of Canadians with IDD. This study was conducted with the understanding that caregivers of Canadians with IDD represent a distinct population who provide ongoing complex care throughout the lifespan [47]. As a result, these individuals may have a unique experience relative to caregivers of other populations. Herein, this study extends previous research-agenda-building efforts focused on people with IDD [48] and responds to calls for a national caregiver strategy [3]. Below, the key findings are discussed, including gaps in the caregiver well-being research landscape that must be addressed in Canada.

Some of the priority areas identified in this study, including “*systems-level programs and services*”, “*caregiver capacity building*”, “*general health promotion*”, “*mental health promotion and mental healthcare*”, “*care and care planning along the lifespan*”, and “*social inclusion*”, have already started receiving some research attention. For instance, two recent studies [29,49] highlight the importance of research to explore longstanding issues with funding and accessing publicly-funded community-based programs and services for caregivers. There have also been calls for theoretically grounded caregiver capacity-building interventions that enhance knowledge and skills to manage caregiving responsibilities [50]. Importantly, research on caregiver capacity-building interventions must consider the multi-layered, complex, dynamic, and relational context in which caregivers and their care recipients operate. Our findings suggest that this work needs to extend to address multiple dyadic health behaviors (i.e., the pursuit of healthy living by two individuals in a socially significant relationship, such as care recipients and their caregivers [51]). Further, identifying intervention mechanisms of action, and understanding under what circumstances such interventions work and for whom is warranted. Related to intervention mechanisms, researchers need to generate knowledge about the effect of social, economic, and environmental determinants of health on outcomes for caregivers. Caregiver-targeted public and private programming and services focused on promoting physical, social, and mental health further require evidence-based tools for ongoing assessment and monitoring of these outcomes along the lifespan. Here, researchers may want to leverage the power of technology tools to support wide-reaching dissemination and implementation efforts.

Another avenue for research is related to examining social structures and dynamics, including how social networks of people with IDD and their caregivers work together to promote their collective well-being. Characterizing the size, composition, and impact of social networks on health and well-being can help to identify “missed opportunities” for improving social connectedness and quality of life [52]. Generating this knowledge can also support the characterization of sustainable caregiving situations and distinguish them from those more precarious. This represents a paradigm shift to expand the scope of health (including rehabilitation) research and practice [53] to focus on supporting and assessing the collective well-being of caregivers and care recipients with IDD.

Of note, identifying these areas as top research priorities in this study reflects the substantial impact that caring for individuals with IDD can have on families, health and social care systems, and society [3,54,55]. However, the findings may also indicate a failure to implement existing local and international caregiver research. Indeed, across the globe, recent and growing attention to the public health crisis of family caregiving across many populations with chronic disease is leading to translating beneficial caregiver programs into community-based services [56]. Stakeholders in this study recognize the importance of concerted, ongoing research efforts to enhance the well-being of caregivers of Canadians with IDD. Research on the priorities identified in this study needs to build on existing promising or proven initiatives for promoting caregiver well-being.

Other priority areas that require attention include “*equity and diversity in care provision*”, *models of care*”, and “*balance of formal and natural community-based supports*”. Regarding equity and diversity in care provision, these constructs continue to be insufficiently addressed in general health [57,58,59], rehabilitation [60], and family caregiver [61] research in Canada and globally, creating barriers to equitable health outcomes for caregivers with intersecting identities. Our findings further support the need for caregiving research that uses an intersectionality approach to examine how multiple dimensions of diversity interact in the context of power and social inequities. Such research would generate a deeper understanding of caregiving and help disrupt the dominant narrative—incorrect/negative assumptions about individuals with IDD and their caregivers embedded in policy, funding, and programs in the Canadian context [62]. Researchers must identify meaningful ways of engaging socially marginalized caregiver groups, who are primarily underserved by healthcare systems, have limited access to services, and likely have the most to gain clinically from interventions. Only by considering the heterogeneity of caregiving experiences can findings with generalizability and applicability across a diverse range of caregiver groups be generated. This is a critical first step to addressing existing disparities in care, where research findings can be used as advocacy for policy and legislation that genuinely addresses the complexity of caregiver needs.

Related to models of care, the nature and quality of care provided to caregivers of Canadians with IDD, by whom, when, in what settings, who pays, and who benefits, must be compared and evaluated. The appropriateness and efficiency of eligibility criteria used by existing models of care (i.e., criteria caregivers must fulfill to ensure they can access funding for services for their care recipients and the impact on caregiver well-being) must also be evaluated. For instance, while Canada has provincial, territorial, and federal policies related to unemployment, family and children, and disability, our participants highlighted that many of these benefits remain unavailable to caregivers of people with IDD who are not currently employed or seeking employment. There is a critical need for our health and social care systems to value family caregivers and to identify creative solutions to support these individuals. Appropriate indices of the utilization of such support structures should be assessed to generate evidence of cost-effectiveness. Some of this work is being conducted internationally and in other caregiving populations, but it needs to be extended to IDD. For instance, Colorado, US, established an innovative paid family caregiver model of care within Medicaid that allows family caregivers to be paid for managing children with medical complexity [2]. As part of this model, formal skills training is provided for the family caregiver to become a certified nursing assistant. The pay for family caregivers who serve in this capacity is no different from a traditional certified nursing assistant, and there is no limit to the number of hours that family caregivers can provide care for their own children. Although this model of paid family caregiving is not without challenges, it can help eliminate the unpredictability of traditional homecare services and provide options and flexibility for family caregivers to care for Canadians with IDD successfully. An example of an innovative model of care in Canada is the newly developed integrated stepped-care model at the Hospital for Sick Kids in Toronto [22]. This model consists of a collaboration between a psychiatrist and a pediatric team managing children with medical complexity and embeds support for caregivers within a public-funded healthcare system. While there are promising practices for children and transition-aged youth with IDD, there is much to learn about supporting families along the lifespan, especially in adult health and social service models of care.

Although the balance of formal and community-based natural supports did not achieve consensus, based on our a priori criteria, this area was included as a priority for research for a couple of reasons. First, even though many people with IDD use more natural (facilitated within the community) than formal (government-funded) supports, most of the emphasis of funding and research is on formal support [63]. Consequently, the voices of family caregivers are often diminished, side-lined, or excluded from spaces where their contributions are critical and irreplaceable by the formal support system. Going forward, researchers must explore how best to strengthen natural supports while considering the individual with IDD and their family’s unique strengths and needs. Second, research should focus on integrating relevant formal and natural supports to benefit multiple dimensions of caregiver well-being. By doing so, researchers can generate information that will allow each family affected by IDD to achieve the right balance of support as their needs and circumstances change over the life span [64].

As with research agendas in general, these directions are not meant to be exhaustive but rather illustrative of key potential opportunities and ideas that may have relevance to broad stakeholder groups beyond our “local Canadian” context, particularly in countries with similar support infrastructures to Canada (e.g., UK and USA). The next step in this caregiver well-being research program is to develop specific research questions to be addressed within each of the priorities. Efforts are underway to develop a Caregiver Wellness Research Collaborative (CARE-Co) housed at the authors’ institution. Because the scope of the work can be broad and necessarily interdisciplinary, CARE-Co will utilize inclusive principles and experience-based co-design approaches [65] to bring together different perspectives and disciplines, including researchers, clinicians, and family caregivers across Canada, to facilitate partnerships to design research studies related to the priority areas identified in this study. This is consistent with a “Nothing about us without us” and social justice approach [66] to empowering families of Canadians with IDD. Part of this work requires situating the emergent research studies and questions within existing theories/conceptual frameworks. For instance, the ethics of care framework [67] is based on the idea that society is embedded in nested dependencies. In this way, caregiver well-being is never entirely independent of the well-being of their care recipients. An ethics of care approach also underscores the responsibility to be bound by the connection to and an understanding of the needs and wants of the other. This framework could ground an exploration of how best to balance formal versus natural supports and the centrality of family and other natural supports found within the community. Further, work grounded in the systems theory [68] could explore the impacts that optimizing disability-related support for families of people with IDD (inclusive of the person with IDD) will have within a broader, normative system in Canada.

### Strengths and Limitations

A strength of this pilot exploratory Delphi study is the inclusion of broad stakeholder groups, most of whom were caregivers of Canadians with IDD, to generate priorities for research focused on enhancing caregiver well-being. Additionally, there was a high level of participant retention between the two survey rounds. Although the Delphi technique relies on opinions, it uses an empirical method to determine consensus. Thus, the research priorities in this study were determined empirically rather than through meetings or discussions.

However, several limitations warrant consideration. Collectively, the issues highlighted herein potentially limit the generalizability of our findings. First, although our final sample size was higher than the lower limit threshold of 12 participants in some Delphi studies [36], heterogeneous groups (e.g., stakeholders from different disciplines) of approximately 30 participants are thought to provide a range of perspectives and improve the reliability of the findings [39]. Given that the composition of participants may affect the results obtained [35], future studies may want to confirm our preliminary findings using a diverse and larger pool of pan-Canada participants for a more representative group of research areas for prioritization. Second, we must acknowledge the inherent bias relative to the recruitment approach used in this study and the resulting composition of our sample. This study enrolled stakeholders who volunteered and expressed interest in participating in the survey. Although the practice of offering payment to individuals to compensate for their time and burden of participation in clinical research is widespread and longstanding [69,70], it is possible that receiving payment may have influenced stakeholder selection. Further, stakeholders were predominantly female, which may reflect the female bias in the caregiving role more broadly [71]. It is possible that the higher education of our stakeholders facilitated their ability to articulate the complex issues affecting caregiver well-being, engage in identifying research gaps, and generate and provide a rationale for priorities. Although most of the stakeholders in this study were caregivers with lived experience, there is a possibility that the high level of education led to the sharing of highly theoretical knowledge from stakeholders’ areas of specific expertise. The study design and how questions were posed may have been inaccessible to individuals with less comfort with using technology (online surveys) and those with less experience, comfort, and/or confidence engaging in these conversations. In addition, despite a comprehensive recruitment strategy, there was relatively limited participation of some stakeholder groups. The reasons for declining to participate are unclear. However, it is possible that navigating the overwhelmed health and social care systems in the current post-COVID-19 pandemic era may have affected the ability of potential participants to complete this study (additional time constraints, unpredictable changes in roles and responsibilities, etc.). These individuals may have perceived participating in research as an additional burden and chose to opt out of the present study. Finally, our decision to stop the Delphi process after the second round precluded us from determining whether stability in our priority topics had been achieved (i.e., consistency of responses across multiple rounds) [72]. However, multiple rounds of questioning can result in participant fatigue and attrition [73]. Consequently, two-round Delphi surveys have been used successfully to develop recommendations in previous studies in the field of health and social care [74,75,76,77], and our methods are consistent with the existing literature. Further, the high level of consensus achieved for all the topics in round 2 suggests that this is likely a minor issue.

## 5. Conclusions

Although preliminary in nature, the list of research topics identified in this study can inform a framework for an ongoing research program focused on improving the well-being of caregivers of persons with IDD. Including caregivers, clinicians, academics, and organizational representatives incorporates perspectives from front-line and support workers and individuals from academic communities with the necessary knowledge and skills related to research approaches and methods. By using this research agenda as a basis for future research, the efforts of the scientific research community will be more focused, intentional, and accountable in addressing the wellness needs of caregivers of people with IDD.

## Data Availability

The data are contained within the article and supplementary materials.

## Supplementary Materials

The following supporting information can be downloaded at: https://www.mdpi.com/article/10.3390/ijerph20227072/s1, Figure S1: The distribution of responses according to the stakeholder group; Table S1: Potential research areas/topics in round 1.
