# Supplementary material for: Identifying Research Priorities to Promote the Well-Being of Family Caregivers of Canadians with Intellectual and/or Developmental Disabilities: A Pilot Delphi Study
Source: Int J Environ Res Public Health. 2023 Nov 16;20(22):7072. doi: 10.3390/ijerph20227072 (PMC10671217; doi:10.3390/ijerph20227072)
Supplement: Supplementary file 1 [file ijerph-20-07072-s001.zip › ijerph-2639009-supplementary.pdf]

Table S1. Potential research areas/topics in round 1.

Item No.	Topic Listed by Participant	Participant Explanation
1	Community support	To create a wraparound support system in neighbourhoods that consists of both formal and informal support.
2	Overcoming systemic barriers	We can’t get adequate services or support (for example, a 160 year wait list for a group home placement for our loved one) and we experience systemic and interpersonal violence from those we do interact with.
3	The impact of aging on the neuromuscular components of cerebral palsy	Anecdotally, we see a lot of Cerebral Palsy cases with extreme rigidity and rapid functional decline beginning in later middle age (late 40s to 60s) that does not seem to be explained by disuse or other factors. There is not a lot of research in the features of Cerebral Palsy in older adulthood. Caregivers have little resources other than just providing more support.
4	The most effective strategies a caregiver could use to effectively support with loved one with neurodegenerative conditions	Caregivers aren't doctors, though we have rich experience with neurodegenerative conditions. We develop strategies as we go, and families share with other families. Yet, having a guidebook or tip sheets of the most effective strategies, based research with medical experts and family caregivers would be a Godsend.
5	Inclusion	Most programs, support, housing, care in general, medical care is not inclusive. They segregate people with disabilities and constantly push them to the margins of society. If we are to implement any effective measures, they must concern society. Every human being counts!
6	Physical support	Many folks living with a neurological condition requires ample physical support and often this is left to the individual doing the caring. Often in the population I work with, careers are also aging and have struggles of their own. Education around how to best help their loved one with personal support, mobility challenges, and equipment needs would be beneficial. Also, how to access support that could be available in the community- both private and OHIP covered.

7	Systems coordination	Often, caregivers access support (for themselves and for their loved ones) across various inefficient and siloed health and social service systems that never seem to talk to one another, increasing stress and burden on families.
8	What tasks and responsibilities caregivers are doing, and how often	There is a lot of literature on caregiver stress and burden, but little research on the factors that lead to this stress and burnout.
9	Supportive housing solutions	As we age, our adult children who live with their parents will have nowhere to live when the parents are either too feeble to care adequately for them, or when they die. Last I heard, there is a 25-year wait list for congregate care housing.
10	What does the care recipient want or need?	A lot of the time, we forget what the care recipient needs or wants, such as a day program, etc.
11	Independence	Having the patient learn from a young age how to become more independent in all areas of their lives.
12	Social justice	Everyone, including those with developmental differences, deserves to live a life of dignity where they are safe, secure, loved and have access to all the services and support needed to live a good life.
13	Mental health	Frequency of co-occurring mental health conditions and better equipping clinicians to support individuals with both autism (IDD) and poor mental health
14	Transition planning	*No description was provided.*
15	Education for caregivers	Learning what education is required for caregivers at the time of diagnosis? Exploring how the knowledge to provide care changes as a child grows or as a degenerative condition progresses. Understanding which team members deliver which types of education? How can we ensure that all caregivers are receiving the same basic education?
16	Personal support workers for caregivers	I believe Caregivers need to have a form of a personal support worker who provides support outside the traditional duties of providing care. For example, caregivers need support in keeping their environment and spaces clean, because they are focused on caring for their loved ones. Support with light household duties such as cleaning the kitchen counters and dusting in the kitchen, washing floors, and cleaning the kitchen, bathrooms, and living spaces. Support with laundry, grocery shopping, and preparing food for the week would also be instrumental.

		Caregivers are very overwhelmed with caring for their loved one(s), and when you add in daily chores, it's overwhelming.<br><br>If there could be funding for this, I would recommend once-a-week support for the caregivers.
17	How to successfully access and benefit from adequate, equitable, and timely healthcare and social services	Investigate how those who have benefited from adequate (getting the right service needed) in a timely manner (within a time frame that made a positive impact on their need) and evaluate how they navigated the system (who helped them, what helped them, what path they took and the steps etc...). Comparing this to those who have not been able to access adequate and timely services to better identify the gaps where planning, priorities and policy changes can be better targeted. Depending on the local system, healthcare and social services may be two separate topics as the systems do not always follow the same pathway.
18	Stress	The impact of stress.
19	Quality of life	Without a quality of life, persons with neurodevelopmental or neurodegenerative conditions will likely die earlier than those without these conditions.
20	Complex medical needs being met in the community	More children with complex medical needs are surviving into adulthood, yet there is no support for those individuals and families. We need to make it a priority to create a system of support for adults with complex care needs.
21	Trauma	Caregivers are experiencing trauma from our loved ones, alongside our loved ones (as they experience interpersonal and systemic violence), and as caregivers. This is intersectional (beyond disability). What are the effects, and how can they be supported (without perpetuating individualized strategies)?
22	Predictive factors for falls and mobility decline in individuals with an intellectual disability	The most predictive factor of whether there will be falls seems to be if there are already falls... it would be nice to catch some of these (no pun intended) before they hit the ground at all.
23	Research on effective communication techniques for caregivers of people with neurodegenerative conditions	People with neurodegenerative conditions have difficulties in communicating. This makes caregiving more difficult. Researching tools, technologies and strategies to make communication better would be great.

24	Quality of life for care recipients. Full citizenship and engagement in society and communities.	Housing that allows a decent, safe and independent life, and employment that is real, meaningful and adapted.
25	Emotional support, social support, and self-care	Helping care partners navigate the challenge of finding time for themselves. Improving access to support groups, mindfulness programs, or yoga. Having opportunities to talk to people who are going through the same experience is essential to being a support person. Education for care providers to understand the importance of looking after themselves while helping their loved ones.<br>Including the importance of staying healthy, sleeping enough, relaxing, finding joy in life, exercising and trying to keep up with friendships and family relationships, hobbies, etc.
26	Recognition of the value of caregiving in society	Caregiving is critical to our society but, in most cases, undervalued and underrecognized. We need to better understand the value (e.g., put financial terms on it for those who think in that way, communicate other values in ways that would resonate with the receiver of the message), then take action to ensure that caregivers and caregiving are better valued and recognized in our society.
27	How caregivers and care recipients make decisions about seeking help from others	There is literature about caregivers not using respite services and other supports, but I haven't seen anything that explores their decision-making process to not use services. If we don't know why they don't use the existing services, we can't change anything.
28	Adequate financial support from the government	Caregivers need resources to support their care recipients who may be living below the poverty line.
29	What do caregivers struggle with?	What tools can be developed that would support caregivers, and why is this support needed?
30	Friendship	Helping each client learn where to find friends, and how to make real long-lasting friendships, and not just have paid help in their lives.
31	Adequate salaries for paid caregivers	Sadly, care for individuals who are elderly, infirmed and/or disabled is under-valued as evidenced by the low pay given to those working in this field. Many of the people working in this field are newcomer Canadians and/or women. The care provided to others is not recognized as a bona fide profession worthy of appropriate remuneration that, in turn, would generate professional pride and ongoing development.

32	Aging supports for adults	As our loved ones age, so do we. There is a chronic fear of "what happens when I'm not here??" or "what happens when I become too old and unwell to care for my loved one?"
33	Mental health	Exploring mental health support options, which is especially relevant post-pandemic.
34	Developing comprehensive caregiver support guidelines	Having caregivers identify the areas of their lives where they need support, and developing guidelines for the various aspects of caregiver health that need to be assessed and addressed by a healthcare team.
35	Caseworkers for caregivers	As a caregiver, you begin to question the status of your own mental health. You find yourself thinking negatively, everything looks overwhelming, and you're tired all the time and not reminded that your self-care is important. All the focus is on your loved one, and the caregiver is not taken care of by any services.<br><br>A check-in from a caseworker by Zoom or by phone could help keep the caregiver on track, and remind them of their basic needs, e.g., eating, sleeping, and exercise, caregivers need to be reminded.<br>Who knows how many caregivers are developing other illnesses due to the stress of caring for their loved ones?
36	How to access adequate, equitable, timely, affordable, and sustainable residential supportive services	Investigate the paths to successful residential supportive services. Compare the paths which encounter barriers and identify the gaps within the system in accessing residential support services. Evaluate the current models and tools that ensure adequate and suitable housing options that meet the needs of the individuals and of their families. Evaluate the impact on individual and family life where experiences in accessing supportive residential services were positive versus negative. Based on findings, develop a tool that can help individuals and families be properly assessed for needs and priorities and how to navigate housing services.
37	Career choice	Exploring why people find themselves in caregiving roles.
38	Housing	The right kind and type of housing is essential to everyone, particularly for individuals experiencing biopsychosocial changes such as health concerns. We need to be planning to better fund, build, and support dynamic co-living spaces and communities where all people can engage with their lived environments (e.g., co-shared spaces while maintaining privacy), car-free with well-supported public transportation infrastructure, affordability regardless of income...

39	Galvanizing community support for caregiving families	Organizing neighbourhoods to provide friendship and support for families who are supporting sons or daughters with disabilities.
40	Harms of improper wellness initiatives	Caregivers are berated with programs and workshops that individualize how they think. As taught by ACT, mindfulness and self-care are patronizing and can do more harm than good for those experiencing trauma. How do we expose the myths and shift focus from toxic positivity?
41	Determinants of dementia-like decline in individuals with intellectual disabilities	When considering cognition and functional abilities, is there any way to predict issues earlier (or perhaps even prevent and ease caregiver burden?)
42	Researching how to diffuse anger and agitation in people with neurodegenerative conditions	A very difficult situation facing caregivers is dealing with anger and agitation in people with neurodegenerative conditions. Research into effective ways for caregivers to calm the situation would be great.
43	Medical research	Medical research directed to help find therapies and medications for people with neurodevelopmental and neurodegenerative conditions.
44	Navigating the healthcare system	I think some people are unsure of how to find help, and how to ask the questions they need to when they are in clinic appointments. I wonder if arming people with strategies to ask good questions, to advocate for themselves and their loved ones in a non-threatening way and giving them a map of where resources exist could be helpful. Many clinicians themselves can feel rushed and so busy that it may come across as a lack of empathy. Questions get forgotten, people feel dismissed and don't know how to keep track of all that is coming their way.
45	Caregiving with an intersectional lens	Much of the research to date in caregiving focuses on the experiences of white, middle-class women. Much more work needs to be conducted to understand caregiving from the perspective of persons who experience layers of marginalization.
46	Caregiving experiences of people who identify as LGBTQ2S+	There is very little literature on caregiving issues among non-heterosexual couples and chosen families. This is a significant gap in the literature.

47	Researching strategies for reduction in self-injurious behaviors	Our system is woefully inadequate in providing information and help. We need more support rather than medication and languishing in a psych ward.
48	What other education can be provided to caregivers?	What types of workshops or courses can be provided to help caregivers
49	Teaching life skills to care recipients	Helping each care recipient become the absolute best person they can be while focusing on "normal" important things such as learning gratitude, kindness, etc.
50	Adequate training for paid caregivers	Kindness and compassion are important elements of care for all people, including those with disabilities, but these elements alone are not enough. How do paid caregivers understand their role in supporting individuals with disabilities in ways that give their lives meaning and purpose? Additionally, people with disabilities are not a homogeneous group, but rather each person possesses unique and complex features that are expressed as gifts as well as challenges. How are paid caregivers trained to acknowledge and adapt their approaches to care and support in a way that recognizes and respects this diversity?
51	Physical health	There is a known higher incidence of comorbidities; how can we support caregivers in providing care for complex conditions?
52	Physical health supports	People with these conditions have varied and specific needs which may not be supported in the current/future healthcare crisis.
53	Availability of supportive services to assist caregivers of persons who have neurodevelopmental or neurodegenerative conditions in Canada	What supportive services are available to caregivers in rural versus urban settings? For low income versus high income? What is the availability of home care and personal support workers?
54	Buddy system for caregivers	Create funding for a buddy system. Match up with like-minded people who are in a good place in their journey to support others. Not a volunteer system, put a value to supporting caregivers. People will stay and support you when you put a value on this service. The buddy system could be a once-a-week check-in by phone, a coffee meeting to get the person out of the house, or a walk-through nature. If we support our caregivers, there could be fewer hospitalizations from burnout and illnesses due to stress.

55	Employment and unemployment support services	Investigate the pathways of those successful in employment and unemployment support services and steps that lead to suitable, equitable, safe and sustainable positive outcomes vs. those faced with barriers and gaps. Evaluate existing employment and unemployment support models for efficacy and accessibility. Unemployment supportive services, which may pertain to many individuals in this population, is a supportive measure that is equally significant as employment as it impacts their financial and social well-being (ensuring financial stability and resources).
56	Effects of the COVID-19 pandemic	Research about burn-out, sick leave, and other effects of caregiving for a loved one during the pandemic.
57	Technology	Technology that drives innovation and implementation is essential to supporting changing lives due to neurodevelopmental and neurodegenerative conditions. We need to be encouraging and supporting designs that utilize human capital.
58	Enabling active retirement for parents of children with disabilities	This priority topic relates directly to independent living for disabled persons. Families want to retain a key role in the life of their loved one with disabilities, but they want retirement and emeritus status. An advisory status.
59	Toxic white (cis-hetero) femininity	Despite statistics that say men are 40% of caregivers (not sure of the data on two-spirited, trans and non-binary individuals), women dominate the discourse, space, research, support groups, etc.<br><br>What can be done to educate professionals and caregivers alike on the violence they perpetuate?<br><br>Whiteness and CIS heteronormativity also dominate these spaces.<br><br>What impact has this had on data, on the health of those excluded? How has it impacted our caregiving experiences situated in our larger life stories?
60	The impact of activities of daily living and functional routines on strength maintenance or loss	Over 2 years of the pandemic shutdown, we've noticed huge and rapid changes in abilities in people who really weren't terribly active, to begin with, but I'm wondering if just those simple acts of putting on pants more often add up more than we thought it did.

61	Community support for people with neurodegenerative conditions	When it is a neurodegenerative condition, very few people want to help you. Families and friends abandon you, as it is not fun dealing with these conditions. It's distressing because it impacts the brain. The so-called "village" disappears. Please research what community supports are out there to support people with neurodegenerative conditions and caregivers. We need our village back - or to create a new one to help us in this difficult journey.
62	Support for the caregivers of people with neurodevelopmental and neurodegenerative conditions	Most of the time, caregivers and families have raised and supported people with disabilities. The toll on the care provider's health, mental health and social lives is so intense and so deep that they need to be supported and cared for.
63	Education	I think educational resources for care partners about their loved one's disease are very important, too. The type of care needed can shift significantly as the disease progresses, and although not everyone follows the same path with their disease, it is important to plan for the future. Living arrangements may need to be shifted, additional support arranged, or even assisted living may be required.<br>Understanding symptoms can perhaps allow someone to be more empathetic and patient. Care partners can also educate their loved ones and help them to take an active role in their treatment.
64	Priorities of carers and care recipients	We should focus research to understand what carers and care recipients need and believe is important.
65	What practical training caregivers want and need, and how this training can be best provided	Caregivers do a lot, and they often take on new responsibilities gradually with little formal training to do what they are doing (ranging from cooking, cleaning, money management, system navigation, medication management, etc.). How would they prioritize their training needs?
66	Research community involvement and acceptance of people with neurodegenerative and neurodevelopmental disabilities	It takes a village to raise a child, we need acceptance for our loved ones.

67	Types of care	What are all the different types of care that are provided in Ontario to support people with neurodevelopmental conditions?
68	Research	Helping each client find the latest information to help them compensate for their specific needs, issues, and illness at a level they can comprehend and following through 1-2 x year on a continual basis.
69	Adequate support for families caring for loved ones with disabilities	Although funding and services to support families with loved ones with disabilities vary from province to province and region to region, many of these families face additional financial costs that others don't. This needs to be adequately addressed in the form of universal benefits for those families caring for loved ones with disabilities that continue throughout the lifespan, considering changing needs over time.
70	Funding for services/support	You can't safely have autism without having an endless source of finances. Nothing is covered, especially if your loved one has a developmental disability without an intellectual disability.
71	Respite options	Currently, there is insufficient funding and limited options for respite care.
72	How prepared are caregivers for coping with the degenerative condition of a loved one?	How do caregivers set expectations for themselves and for their loved ones? How do they cope with a sudden or gradual change in status?
73	Tradespeople to support caregivers	As caregivers support their loved ones, and there is a financial increase that comes with this, other aspects of life are neglected. If people live in a house, there is upkeep and maintenance, and often, there isn't enough money for this. It would be helpful if there was an organization of tradespeople who offer a sliding scale for caregivers with loved ones who are disabled. For example, someone to cut the grass, clean the windows, repair plumbing or electrical light fixtures, some basic jobs that can help caregivers feel like their place is not falling apart. This is especially relevant as more caregivers are part of an ageing population.
74	Transition support	Investigate and identify support measures, tools, strategies and pathways that lead to positive outcomes during various transitions throughout the life span. Hospital life- to home, home to school, elementary to high school, college, vocational, recreational, etc. Transitions from various supportive healthcare or social services, personnel, facilities or programs. Transitions that relate to employment, unemployment, and residential (home to group home or apartment, long-term care facility, senior long-term care facility). Identify

		and evaluate tools that exist and/or develop tools to test transition tools and measure their effectiveness. There are very few transition tools developed or used. There is a need for training for families, caregivers, teachers, service providers and facility employees to understand the need and strategies for positive transition measures (and to understand the negative impact when it does not exist to support an individual who requires it).
75	Sleep	See if there is a higher rate of sleep deprivation amongst caregivers.
76	Economics	We need to vastly improve the financial lives of those living with neurodevelopmental/neurodegenerative conditions, whether this be funding in all areas of people's lives, enhancing the lives of those providing the services and supports, or improving people's living conditions.
77	Contribution	We need to support and enable the political and civic participation of people with disabilities. They can become climate activists, politically engaged, etc. This needs to be a priority.
78	Silos	Neurodevelopmental disabilities are siloed off from medical and mental health systems. This has created innumerable problems, and we and our loved ones do not receive a consistent standard of care. How and should we create an integrated and inclusive system (and is that even achievable), or how can we fix the systems that exist?
79	Attitudes on health promotion in DD	How much do adults with DD or dual diagnosis understand about the relationship between what they do (exercise, nutrition, mindfulness, healthy social connections, etc.) and how healthy they are?
80	Communication and training for caregivers	For example:<br>How can caregivers ask hard questions when they might not like the answer?<br>How to advocate for someone who cannot advocate for themselves?<br>How to communicate with someone who is living with a progressive disease (e.g., dementia, aphasia, deafness, cognitive changes)?<br>How to ask for help and share the truth of the burden that caregiving can sometimes impart?
81	Balancing formal, natural, and informal supports in caregiving	Often, formal support is given priority in our society. I want to understand the aspects of natural and informal support in caregiving; what do people enjoy doing with their loved ones? How could caregivers be better supported to offer or access informal and natural care to the extent that they want to; and how that can be balanced or supplemented with the

		appropriate amount of formal support to help the family (inclusive of the loved one needing care) have a good quality of life?
82	Coping strategies for "successful caregivers"	There are caregivers who seem to be able to take on a lot and who are able to manage it all well. What are they doing differently? How can we learn from their successful management?
83	Researching technical advancements	Are there communication tools, or apps that could be supportive for caregivers; especially tools that are not cost-prohibitive?
84	Salary	How much versus how happy are caregivers in their roles as carers? How does private versus public care differ?
85	Real solutions	Helping each patient or client find a real solution to everyone's adult life. How they will participate in the same important matters that typical young adults need to figure out.
86	Adequate mental health support for family caregivers of people with disabilities	Many families face incredible barriers in their quest to support and care for their loved ones with disabilities. Lack of acceptance of the value of such individuals and their right to live a good life has a cumulative negative impact on the mental well-being of those who love and care for them.
87	Alternative for applied behavioral analysis therapies	Look at ways to support without trying to change outward behavior. Put research into appreciating and understanding neurodiversity.
88	Meaningful community engagement for care recipients	There are insufficient opportunities for community engagement, and I fear that there will be even fewer opportunities post-pandemic
89	Healthcare utilization of caregivers versus age-matched controls	Identifying if caregivers have higher healthcare needs for their own health could assist in advocating for more supportive programs.
90	Food support	Finding food support funding (e.g., a food bank) and having a program or system that has food delivered to the house; people are limited as to when and for how long they can leave the house.<br>Also, have premade meals funded, perhaps partnering up with a restaurant, and at the end of the night, they could freeze any leftovers, which could be delivered to families in need once a week.

		Most of the time, caregivers are tired and not able to make a balanced meal for themselves or their loved ones.
91	Long-term health care planning and care	Investigate and evaluate tools, strategies and pathways to positive outcomes for aging individuals with needs that are beyond what is typically seen in nursing homes. Many caregivers express great fear and anxiety about long-term plans and care for their loved ones. How to plan, and ensure good care and even elder care (when caregivers may no longer be present). Evaluate current models, facilities and training for caring for aging individuals with specific needs and diagnoses. Best practices and guidance on how to access and navigate the legal realm of caregiving (tutorship, power of attorney, estate planning, etc.)
92	The impact of shift work	Research the effectiveness of shift work - mentally, emotionally, physically and socially.
93	Community building	Without community, we are less. Community = Belonging = Quality of Life = Joy. From creating co-shared spaces like free libraries, food hubs, micro meeting spaces, nature bathing, and more, community is life.
94	Employment opportunities	Everyone needs a job to feel good, to feel important, and to feel that they are contributing to society. We need to find better pathways to employment for disabled Canadians.
95	Compensation for caregivers	As caregivers often cannot work (because staff or paid caregivers do not exist or are cost-prohibitive) or cannot work to the full extent of their capabilities, they are forced into poverty or precarity. Current programs end when our kids are 18, are temporary, or are only predicated on having had a job recently. What would adequate compensation look like for those who are unemployed because they have no other choice but to be unpaid carers?
96	International comparisons of neurodevelopmental supports and research focus	Nordic Europe just seems to be a progressive place to be if you want to manage complex health conditions. Why is that? Why can't Canada adopt policies more like Sweden?
97	Financial support and education for caregivers around how to access funding or assistance with managing funds	Many folks come to my clinic with limited resources. They find that:<br>therapies are expensive, and many lack insurance; medications are expensive; there are tax credits and deductions available but navigating this without assistance can be challenging; balancing work and caring for a loved one is very difficult; the sandwich generation is lacking support to care for both aging parents and children; and many would benefit from learning more about time management skills.

98	Peer support	What is the value in having others with similar experiences supporting each other in the caregiving journey? What can people with lived experience offer to other caregivers that others without lived experience simply cannot? How can meaningful peer support be facilitated? What are the barriers?
99	What do caregivers want and need from members of the formal healthcare team who are also providing care?	We talk about patient- and family-centered care, but it is not clear what caregivers want and need from the healthcare providers who are supporting, treating, and managing the care recipient.
100	Researching how to get loved ones meaningfully engaged in their lives	Caregivers struggle to provide meaning to loved one's lives.
101	Private vs. public care	What are the challenges, benefits, and differences between the services offered publicly and privately in Canadian provinces?
102	Coping	How to incorporate coping mechanisms and real solutions for planning for each individual's future with concrete solutions and helping to put the plans in motion, instead of just making suggestions. A complete follow-through every few years with annual visits to re-assess strategies and solutions for caregivers.
105	Adequate social assistance for adults living with disabilities	Although funding to financially support adults with disabilities varies from province to province, in all cases, it is woefully inadequate.
106	Support with justice and social system	Quiet, well-mannered adults with IDD/DD are welcome in group homes. There is no space, support or respite for those whose frustration is expressed in dangerous or less desirable ways.
107	Employment or volunteering opportunities for care recipients	To promote independence and financial security.
108	Preventing burnout of caregivers	What are some early signs of burnout? How can caregivers be supported before getting to the point of burnout?

109	Caregiver disability allowance	Just like the child tax credit people receive every month, the caregivers could benefit from a monthly cheque to make their own decisions about where they need the support more. Everyone's situation is different. Not all these services may be beneficial to every caregiver. Some people need support in housing, food, or medical equipment, which the government doesn't cover, such as, special shoes, eye care, hygiene products, or vehicle supports.<br><br>Caregivers deserve treats also, maybe a trip to get their hair done, a take-out meal, a plant, or some high-protein meals from the grocery store that are healthy and ready to eat.
110	Caregiver support, respite, training, and resources	Identify measures and pathways that lead to positive caregiver support, respite, resources and training. Compare pathways with those who faced challenges to help identify barriers and gaps within the current system. Evaluate or create a tool to measure the well-being of caregivers, which can be used as a tool for families or care-partners in the community to act when there are risks identified. Often, the individual is the focus of care and support 24/7. The focus on support also needs to extend to those who are providing care.
111	Trauma care	Improve the supports for caregivers
112	Meaning making	Enhancements to people's existing “gifts” with opportunities to realize those gifts provide equity and person-centered existence.

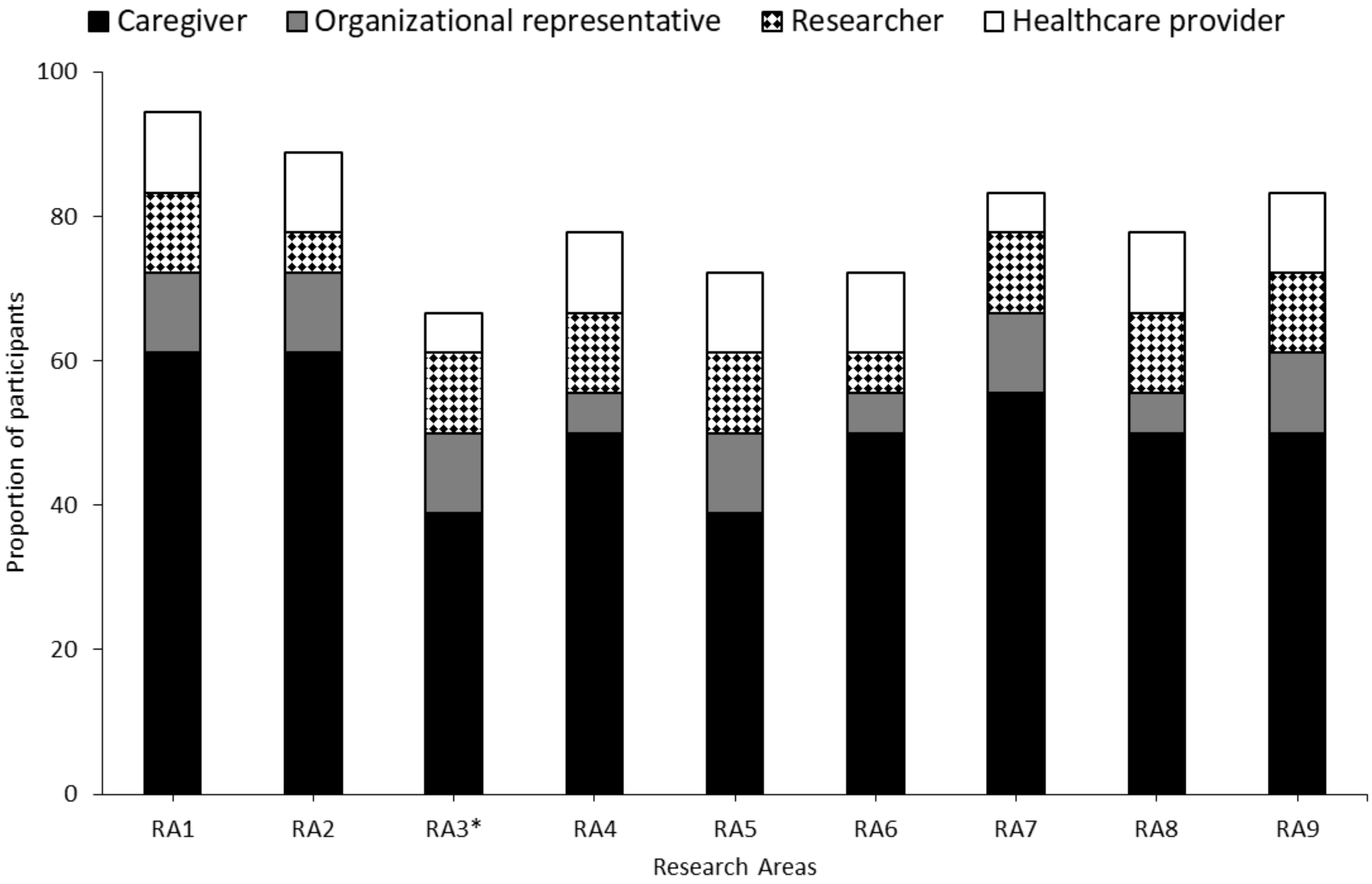

Figure S1. The distribution of responses according to the stakeholder group.
